# Infant Mortality and Infants’ Food

**Published:** 1877-10

**Authors:** 


### Infant Mortality and Infants' Food.

The summer mortality from diarrhœal diseases, according to Dr. Baginsky, has quadrupled in Berlin during the past twenty years; no other cause of death has presented such a marked increase. In seeking for the cause of this mortality, Dr. B. made a series of experiments concerning the comparative merits of the articles of diet which are ordinarily allowed to infants. He experimented with human milk, cow's milk, condensed milk (Swiss), two varieties of farinaceous food, and two specimens of "prepared" infants' food. Each of these articles was exposed to a temperature of 67° for a period of twenty-four hours; he found that the human milk and that from the cow remained almost unchanged, but the Swiss milk and the other foods, although still appearing fresh and wholesome, yet exhibited bacteria in active motion. The reaction of the human milk was alkaline; the cow's milk slightly acid; the foods were strongly acid. After a further exposure of eighteen hours, the Swiss and cow's milk were found to be coagulated, while the four foods were in a high state of putrefaction. The human milk, however, still remained alkaline and almost unchanged. These experiments are interesting as vindicating the high estimation in which the human milk is held; but their great lesson is the necessity of guarding the substitute-food from the putrefactive changes which are set up so rapidly in our hot season. Our summer mean temperature, in 1874, was fully 7° higher than that chosen by Baginsky for his observations. Nicer tests must be applied to infants' foods, including those for acidity and microzymes. The relations of bacteria to cholera infantum have not yet been demonstrated, but their evil influence is suspected. Their coincidence with diarrhœal mortality in the tenement-house district is noteworthy, if we can rely upon the statements of those who state that the air of tenements is polluted, not only by causes from without, such as filthy streets and gutters, but by the very walls and appurtenances within them, which reek with low forms of life in the hot season. Their collected filth then takes on an unseen life and activity, putrefactive germs increase and multiply, float in the air, and, no doubt, attach themselves to, and contaminate the articles of food and drink which lie exposed. So that the increased care with reference to the covering of food, and the keeping of it at a low temperature, assumes a new importance in those sections which Dr. Elisha Harris has styled the "*pauvres faubourgs*" of our cities.—*Proceedings of the Medical Society of the County of Kings.*

